# Real-Time Emotion Recognition Performance of Mobile Devices: A Detailed Analysis of Camera and TrueDepth Sensors Using Apple’s ARKit

**DOI:** 10.3390/s26031060

**Published:** 2026-02-06

**Authors:** Céline Madeleine Aldenhoven, Leon Nissen, Marie Heinemann, Cem Doğdu, Alexander Hanke, Stephan Jonas, Lara Marie Reimer

**Affiliations:** 1 Institute for Digital Medicine, University Hospital Bonn, University of Bonn, 53113 Bonn, Germany; 2 German Center for Neurodegenerative Diseases (DZNE), 53127 Bonn, Germany; 3 Department of Old Age Psychiatry and Cognitive Disorders, University Hospital Bonn, University of Bonn, 53127 Bonn, Germany

**Keywords:** face tracking, ARKit, emotion recognition, real-time, sensors

## Abstract

Facial features hold information about a person’s emotions, motor function, or genetic defects. Since most current mobile devices are capable of real-time face detection using cameras and depth sensors, real-time facial analysis can be utilized in several mobile use cases. Understanding the real-time emotion recognition capabilities of device sensors and frameworks is vital for developing new, valid applications. Therefore, we evaluated on-device emotion recognition using Apple’s ARKit on an iPhone 14 Pro. A native app elicited 36 blend shape-specific movements and 7 discrete emotions from N=31 healthy adults. Per frame, standardized ARKit blend shapes were classified using a prototype-based cosine similarity metric; performance was summarized as accuracy and area under the receiver operating characteristic curves. Cosine similarity achieved an overall accuracy of 68.3%, exceeding the mean of three human raters (58.9%; +9.4 percentage points, ≈16% relative). Per-emotion accuracy was highest for joy, fear, sadness, and surprise, and competitive for anger, disgust, and contempt. AUCs were ≥0.84 for all classes. The method runs in real time on-device using only vector operations, preserving privacy and minimizing compute. These results indicate that a simple, interpretable cosine-similarity classifier over ARKit blend shapes delivers human-comparable, real-time facial emotion recognition on commodity hardware, supporting privacy-preserving mobile applications.

## 1. Introduction

Facial expression is an information-dense channel, from short-lived expressions that communicate affect to stable morphological cues that support clinical and forensic reasoning [1,2]. In clinical genetics; for example, deep learning over face images can surface syndrome-specific facial information and assist expert diagnosis at scale [1]. In forensic settings, facial comparison from CCTV imagery remains consequential yet sensitive to acquisition quality and methodology [2]. However, outside such controlled or expert-driven contexts, continuous and accessible analysis of facial signals is often limited by computational costs, data privacy concerns, and the need for specialized hardware. Against this backdrop, reliable real-time facial emotion recognition on commodity hardware could unlock longitudinal assessment, tele-rehabilitation, and Human–Computer Interaction applications while keeping raw video data on-device for latency and privacy.

Recent advances in physiological signal-based emotion recognition, particularly using EEG, offer a complementary perspective to vision-based approaches. Many current methods focus on improving generalization across individuals by modeling temporal dynamics and reducing subject-specific variability. For example, STRFLNet [3] combines spatial and temporal EEG features to improve emotion recognition across participants, while graph-based domain-adaptation methods learn subject-invariant connectivity patterns [4]. These research insights highlight the potential of physiological modalities and motivate future multimodal approaches that combine camera-based facial analysis with physiological signals for more robust emotion recognition.

Two pragmatic trends make on-device facial emotion recognition particularly timely. First, modern smartphones include depth-assisted front-facing sensors (e.g., Apple’s TrueDepth since iPhone X in 2017) that enable robust face modeling in everyday conditions [5]. Second, widely deployed AR toolkits expose a compact, interpretable representation of facial movement through blend shape coefficients (continuous 0–1 activations of semantically named facial features) at video rate—e.g., ARKit’s ARFaceAnchor.blendShapes on iOS and MediaPipe’s Face Landmarker blend shape outputs [6,7,8,9]. These coefficients can be mapped to the Facial Action Coding System’s (FACS) [10,11] action units (AU), offering an attractive theoretical framework for lightweight, explainable inference.

While deep convolutional models dominate facial emotion recognition research and report strong accuracies on curated benchmarks [12,13], their computational and data demands can hinder deployment in low-latency, always-on mobile scenarios. Moreover, the opacity of large neural models complicates clinical interpretability and on-device validation [14,15,16,17]. This motivates revisiting simple, geometry-aware baselines that operate directly on blend shape vectors, preserve interpretability via FACS-aligned prototypes, and are fast enough for real-time use.

We aim at making the following three main contributions: (i) Describing a fully on-device, interpretable facial emotion recognition pipeline that uses FACS-guided prototypes and cosine similarity for classification, requiring only per-frame blend shape vectors, no server, and no heavy model. (ii) Releasing a measurement protocol using a native iOS app (ARKit + TrueDepth) to elicit 36 blend shape-specific movements and 7 discrete emotions, enabling subject-level standardization and analysis. (iii) Providing a direct human comparison: three independent raters (two psychologists) annotated the same videos, allowing a side-by-side evaluation of machine vs. humans on overall and per-emotion accuracy.

Our results suggest that, for smartphone-based facial emotion recognition grounded in explicit facial geometry, a carefully constructed cosine-similarity classifier is a competitive and deployment-friendly baseline. Beyond establishing a practical floor for real-time performance, this finding complements prior work on ARKit’s measurement fidelity (e.g., gaze estimation accuracy and high sample rates) and encourages broader evaluation of simple, interpretable methods before resorting to complex models [18].

## 2. Methods

This study is designed to evaluate Apple’s ARKit framework’s performance in recognizing basic human emotions and to contextualize its performance with human raters. We hypothesized that cosine similarity using ARKit’s blend shapes in emotion vectors, as a lightweight computational approach, can achieve accurate, real-time emotion classification, surpassing the accuracy of trained human observers.

### 2.1. Mobile Application and Data Collection

A custom iOS application (iOS 16/17) was developed using Swift 5.7, incorporating ARKit for 3D face tracking, UIKit for user interface rendering, and ARVideoKit (Version 1.6.0) (https://github.com/AFathi/ARVideoKit (accessed on 2 February 2023)) for synchronized video capture. The app leveraged the iPhone’s front-facing TrueDepth sensor to record 52 blend shape coefficients per frame. Each coefficient represents the normalized activation (0–1) of a specific facial muscle movement. These blend shapes are consistent with other frameworks (e.g., MediaPipe, Unity) and map [11] onto the Facial Action Coding System (FACS) [19]. Data were stored in CoreData and exported as CSV and MP4 files for analysis. The code for the data recording app is available on GitHub (Version 1.0.0) (https://github.com/digital-medicine/ARFaceAnalysis.git (accessed on 2 Februrary 2026)).

### 2.2. Study Design and Participants

We recruited N=31 healthy adult participants (University Hospital Bonn staff, students, and affiliates), and informed consent was obtained. Participants varied in sex, age, and ethnic background, but none were trained actors. Demographic data, including age, sex, body mass index (BMI), ethnicity, and facial features (e.g., beard, glasses), were collected. The study was approved by the institutional ethics committee of the University Hospital Bonn (015/23-EP).

**Figure 1:**
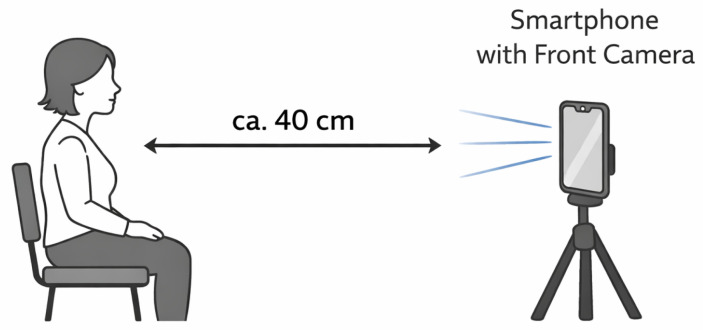
Experimental recording setup.

Each participant performed 43 facial expressions: 36 blend-shape-targeting movements and 7 discrete emotions (anger, contempt, disgust, fear, joy, sadness, and surprise). To minimize external confounding factors, the device was set to airplane mode, Wi-Fi was disabled, and low-power mode was turned off. Measurements were repeated across three independent runs. For each run, the application was allowed to remain idle for at least 5 s to establish a stable baseline and to mitigate potential overhead introduced by the profiling process itself. Subsequently, each recording trial lasted 5 s and followed a standardized sequence: participants began with a neutral facial expression (approx. 1 s), then gradually transitioned to a maximal expression (apex), which was maintained and released within the remaining approx. 4 s. All expressions were elicited via verbal instructions and live demonstrations by trained study personnel. This procedure was applied consistently across all 36 blend-shape-targeted movements and the 7 discrete emotion recordings. To minimize variability, the second batch of participants (N=19 out of a total of N=31) was recorded using a tripod-mounted iPhone 14 Pro at a standardized distance of 40 cm under constant lighting conditions, rather than holding the phone themselves as in the first batch. Each recording was saved as a synchronized CSV (blend shapes) and video file. A schematic illustration of the experimental setup is shown in Figure 1.

### 2.3. Ground Truth Mapping: FACS and EMFACS

Target emotions were defined using the Emotional Facial Action Coding System (EMFACS), which maps FACS action units to canonical emotions. Blend shapes were mapped to action units based on a published guide [11]. For example, browDownLeft/Right corresponds to AU4 (brow lowerer), while mouthSmileLeft/Right corresponds to AU12 (lip corner puller). Table 1 provides the complete mapping of blend shapes to emotions and which action units were translated into which blend shape according to a published mapping [11].

In our recordings, each blend shape corresponds to a specific facial movement or expression (e.g., raising the eyebrows or stretching the mouth). To simplify data collection, we recorded symmetric pairs (e.g., browDownLeft and browDownRight) together by asking participants to perform the movement on both sides simultaneously. Following this approach, we captured all 52 ARKit blend shapes within 36 recordings.

**Figure A1:**
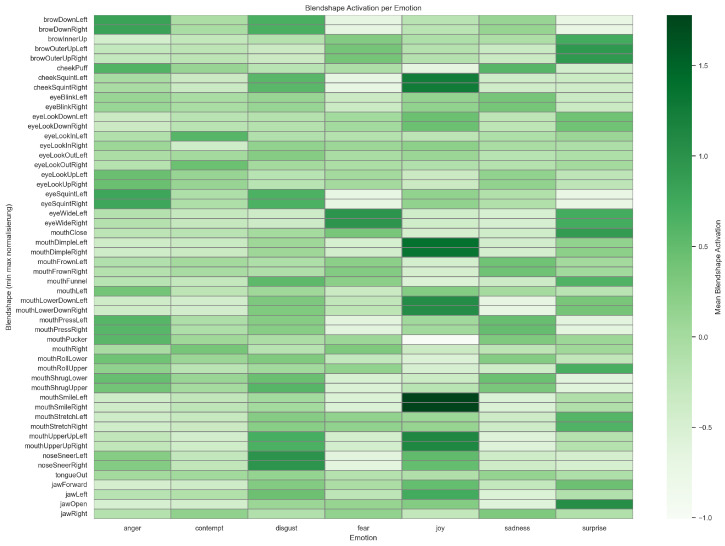
Blendshape activation per emotion.

We additionally explored extending the published ARKit-to-FACS mapping [11] by adjusting the set of included blend shapes, both through analysis of emotion-specific activation heatmaps (see Figure A1 in the Appendix A) and by manually adding blend shapes we hypothesized to be informative. However, these modifications yielded negligible or slightly degraded performance, so we retained the original mapping in all subsequent experiments.

### 2.4. Human Raters

For comparison with ARKit-based classification, three human raters independently labeled all emotion videos (*N* = 217; 7 emotions across 31 participants). Two raters were trained psychologists, and one was a researcher familiar with facial expression coding. Videos were presented in a randomized order, and raters selected the most likely expressed emotion from a set of 7 discrete emotions used in our study.

The raters perceived that some subjects exhibited minimal to no facial expressions in the videos. One subject, in particular, demonstrated a generally flattened affect, potentially indicating a deviation from normative behavior. Additionally, in some recordings, the camera refocused within the first few seconds, resulting in a brief period of blurriness. However, all subjects and recordings have been processed in this analysis as they would be in real-world use cases. We calculated inter-rater agreement using Krippendorff’s α.

### 2.5. Data Preprocessing

Recordings were captured at either 60 frames per second (FPS, 80.67% of data) or 30 FPS (19.23%). To harmonize sampling, 60 FPS sequences were downsampled to 30 FPS by omitting every second frame. This prevented temporal imbalance across subjects without introducing interpolation artifacts.

For each subject, blend shape values were standardized using z-scoring:

(1) $$z=\frac{x-\bar{x}}{\sigma }$$

where *x* is the raw blend shape value, $\bar{x}$ is the subject-specific mean, and σ is the subject-specific standard deviation. This normalization reduced inter-individual variability and emphasized relative activation patterns.

We did not apply a separate baseline correction. Because our analyses relied on cosine similarity between standardized blend shape vectors, the relative pattern of activations, rather than their absolute magnitudes, was the primary signal of interest. Z-scoring already centers each subject’s data around their own mean activation level, effectively normalizing for individual baselines while preserving within-expression dynamics. Moreover, given the brief, discrete nature of our recordings, additional baseline subtraction risked amplifying noise. Thus, z-scoring combined with cosine similarity yielded a stable, interpretable normalization scheme for our analyses.

To reduce onset and offset effects, analyses were restricted to the frames corresponding to the sustained expression phase, excluding the initial neutral segment of each recording. Data analysis was performed using Python 3.12.1.

### 2.6. Blend Shape Analysis

To gain further insights into blend shape behavior during facial movement, we calculated the mean, minimum, and maximum values for each blend shape and compared them. Since some blend shapes represent opposite actions (e.g., EyeLookup vs. EyeLookDown), we further investigated their co-activation patterns by calculating the overlap of data points where both blend shapes were activated:

(2) $$\mathrm{Overlap}=\frac{\left|\{i|x_{i}>0\;\wedge \;y_{i}>0\}\right|}{N},$$

Co-activation of distinct facial regions (jaw, eye, mouth, cheek, brow, nose, tongue) during facial movement was investigated by calculating Spearman’s correlation coefficients between the aggregated blend shapes for each region.

### 2.7. Emotion Classification Approaches

Two computational approaches were tested.

#### 2.7.1. Simple Mean-Based Classification

For each target emotion, we computed the mean activation of all relevant blend shapes (as defined by EMFACS mapping [11]). The emotion with the highest mean activation was selected as the predicted label.

#### 2.7.2. Cosine Similarity Classification

For each frame, we constructed an emotion prototype vector by averaging the standardized blend shapes across all recordings of that emotion. Classification of a new sample was performed by computing the cosine similarity between its blend shape vector *v* and each prototype $p_{i}$:

(3) $$\mathrm{cosine}\left(v,p_{i}\right)=\frac{v\cdot p_{i}}{∥v∥∥p_{i}∥}.$$

The emotion with the highest similarity was selected. Cosine similarity was chosen for its simplicity, scale invariance, and suitability for real-time deployment on mobile devices.

We evaluated several simple, real-time-capable classification strategies that operate directly on standardized blend shape vectors (including mean-based aggregation and distance-based matching). Cosine similarity consistently provided the best performance while preserving computational simplicity. Conceptually, cosine similarity compares the directions of two vectors rather than their magnitudes, making it well-suited for z-scored blend shape data, where relative activation patterns across facial action units are more informative than absolute intensities. This scale invariance reduces sensitivity to individual expressiveness, distance to the camera, and global activation strength, while emphasizing emotion-specific co-activation structures defined by EMFACS. From a deployment perspective, cosine similarity requires only vector dot products and norms, enabling deterministic, low-latency, and energy-efficient real-time inference on mobile devices without model training or parameter tuning.

### 2.8. Evaluation Metrics

Prediction performance was evaluated per emotion and overall. Accuracy was defined as the proportion of correctly classified expressions. In addition, Receiver Operating Curves (ROC) curves were constructed, and the Area Under the Curve (AUC) was calculated for each emotion. Human raters’ accuracies were averaged per emotion and across all emotions. ARKit-based methods and human ratings were compared directly.

To assess the runtime impact of on-device face detection and emotion classification, we conducted a performance analysis using Xcode Instruments on an iPhone 14 Pro. During execution of the recording application, we captured overall system activity, thermal state, GPU events, as well as application-specific CPU utilization and memory footprint.

To benchmark our emotion recognition approach against a state-of-the-art deep learning baseline, we employed OpenFace 3, which includes a CNN-based facial emotion recognition model trained on the AffectNet dataset (a collection of 60,000 images annotated with the emotion categories neutral, happy, angry, sad, fear, surprise, disgust, and contempt).

We processed the same video recordings used in our ARKit-based analysis to ensure comparability. All videos were analyzed offline on a workstation equipped with an AMD Ryzen 9 7950X, 128 GB memory, and an NVIDIA RTX 4090 GPU. For each frame, OpenFace produced predictions for the most likely emotion class, as well as additional facial behavior descriptors including facial landmarks, action units, and gaze direction. The per-frame outputs were stored in CSV format for subsequent quantitative analysis.

## 3. Results

### 3.1. Demographics

Out of the 31 participants, 14 were female. The age ranged from 22 to 87 years, with a median of 28 years and an inter-quartile range of 26–37 years. Ten participants had beards, and eleven participants wore glasses.

Participant BMI ranged from 17.56 to 37.11 (median 22.86, IQR 20.76–25.63).

The study’s participants were predominantly Caucasian. Additionally, the sample included two participants of Arab descent and one participant of Asian descent.

### 3.2. Blend Shapes and Action Units

**Figure 2:**
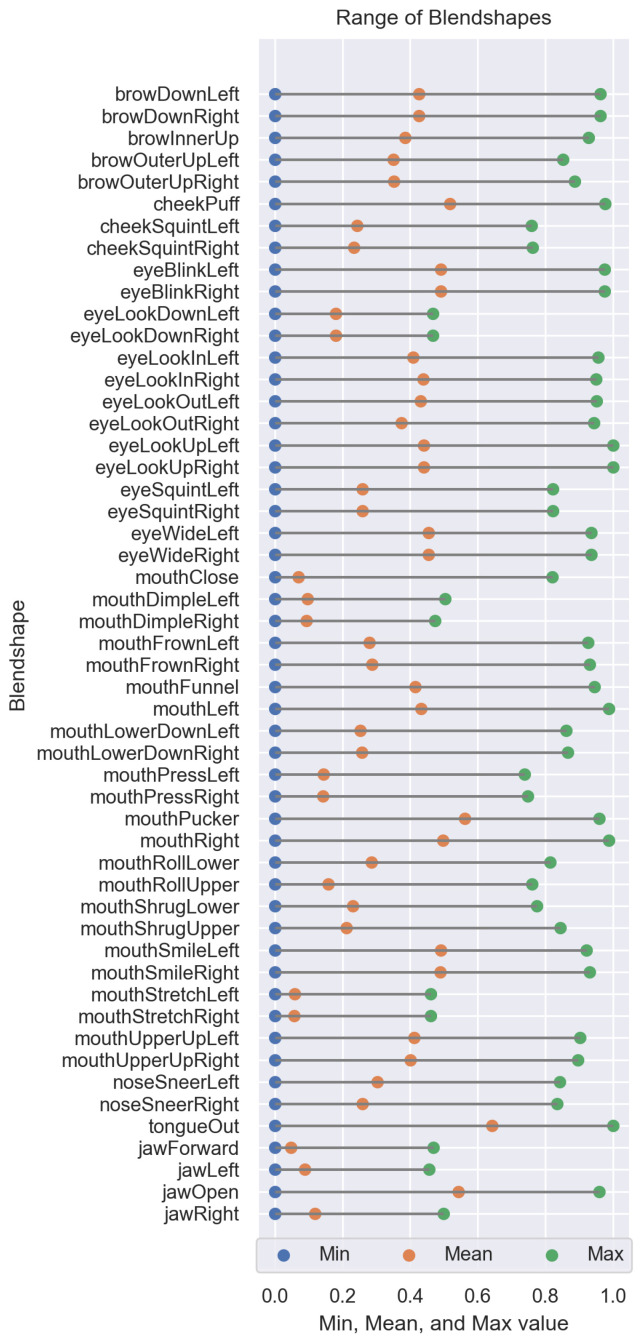
Minimal, maximal, and mean values of all blend shapes coefficients across all subjects.

Figure 2 presents a visualization of the range of values for all blend shapes and all subjects that appeared in our study, displaying the minimum, mean, and maximum values for each. The x-axis ranges from 0.0 to 1.0, while the y-axis lists the blend shapes. Each blend shape is represented by three markers: blue dots indicating the minimum values, green dots representing the mean values, and orange dots denoting the maximum values.

All blend shapes feature a minimum value of zero, which is, according to ARKit (https://developer.apple.com/documentation/arkit/arfaceanchor/blendshapelocation (accessed on 2 February 2023)), ARKit’s facial recognition framework from Apple, representing a “neutral” position.

Certain blend shapes, such as cheekPuff, eyeBlink(Left (L)/Right (R)), mouth(L/R), and tongueOut, exhibit a wide range of motion, with maximum values close to 1.0; eyeLookUp(L/R) stands out, with a maximum value of 1.0. Other blend shapes, such as eyeLookDown(L/R), mouthDimple(L/R), mouthStretch(L/R), jawForward, and jawRight, display a relatively narrow range, with jawLeft being the narrowest, at 0.45. Blend shapes such as eyeBlink(L/R), browDown(L/R), and mouthSmile(L/R), which have corresponding positional counterparts, generally display similar ranges and mean values, indicating consistency across both sides of the face. In our correlation analysis, we observed that these symmetrical blend shapes (e.g., browDown(L/R)) exhibit strong correlations with one another. Additionally, eyeLookInLeft and eyeLookOutRight, as well as eyeLookInRight and eyeLookOutLeft, also demonstrate significant correlation (r > 0.8). Furthermore, we found that mouthPress(L/R) is highly correlated with mouthShrug(Lower/Upper).

The mean values for many blend shapes (17/52) tend to be closer to the lower end of the scale (<0.25), indicating that the maximum values are rarely reached and that a large portion of the data are concentrated near zero. Some blend shapes, such as jawOpen, mouthSmile(L/R), eyeWide(L/R), eyeBlink(L/R), or cheekPuff, exhibit high maximum values and mean values near 0.5, indicating that ARKit can better recognize these expressions or that they are easier for subjects to perform.

Some blend shapes have corresponding opposites. When one activates, its opposite has a neutral (0.0) value. We identified 12 pairs of opposite blend shapes in our analysis (see Table 2).

Although eyeWide(L/R) and eyeSquint(L/R) are not completely opposite to each other, they exhibit noticeable opposite effects.

**Figure 3:**
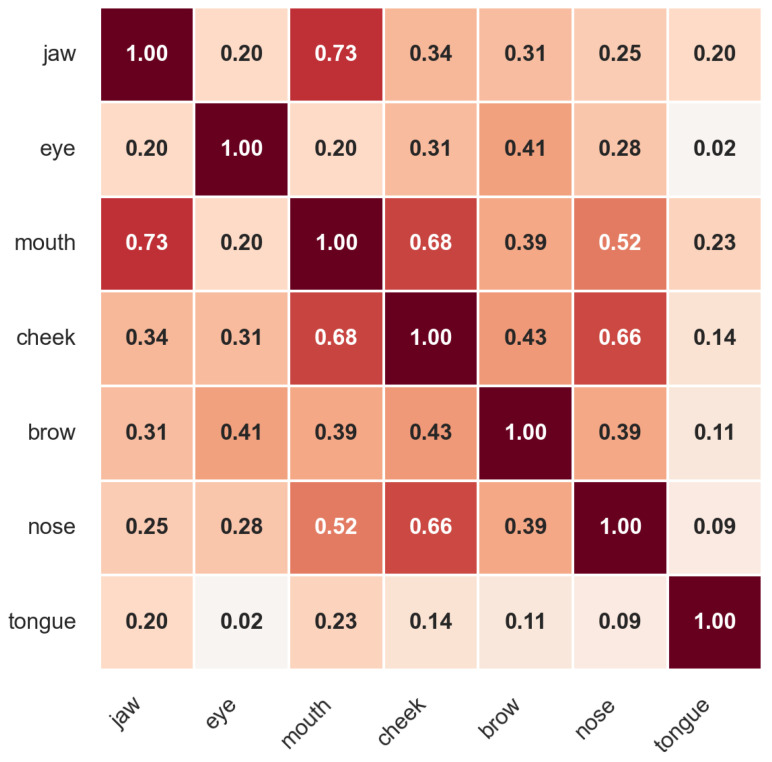
Correlation matrix representing co-activation patterns between facial regions (jaw, eye, mouth, cheek, brow, nose, tongue). A higher correlation indicates that the correlated regions tend to get activated at similar times. All correlations were significant with *p*-values < 0.001.

Finally, when categorized to their facial areas, the following correlations were identified (see Figure 3): the brows are correlated with the eyes and nose, the cheeks are correlated with the mouth and nose, the mouth is correlated with the jaw and cheek, and the tongue is correlated with the jaw. These correlations indicate intricate inter-dependencies among different facial regions and their respective blend shapes.

We investigated the activation of all blend shapes per emotion in a heatmap and explored whether different combinations of blend shape selections could improve the model’s performance, but this did not occur. Therefore, we kept the original mapping based on EMFACS.

### 3.3. Real-Time Emotion Recognition Performance

We evaluated two computational approaches on the same ARKit blend shape recordings: (i) a simple method using the mean of the sum of emotion-relevant blend shapes and (ii) the proposed cosine similarity classifier. The baseline achieved an overall accuracy of 40.18%, whereas the cosine similarity approach reached 68.30%, yielding an absolute improvement of +28.12 percentage points (pp), i.e., a relative gain of ∼70% over baseline.

Table 3 summarizes device-based methods alongside human rater performance. Across the three raters, individual accuracies were 59.66% (R1), 64.75% (R2), and 52.17% (R3), with a mean of 58.86%. The proposed method exceeded all individual raters and outperformed the best human rater (R2) by +3.55 pp and the human average by +9.44 pp (a relative gain of ∼16%).

Although the dataset includes an objectively correct ground-truth label for each video (the emotion that participants were instructed to display), human raters showed extremely low inter-rater agreement (Krippendorff’s α = 0.01). This indicates that humans interpreted the same facial expressions very inconsistently, which is expected for posed, subtle, or ambiguous expressions. Despite this low agreement between raters, individual human accuracies with respect to the ground truth ranged from 52% to 65%. In comparison, the proposed cosine-similarity method achieved 68.3% accuracy, outperforming all three raters and the baseline method (40.18%). Because the ground truth is objective and independent of human perception, the low α value does not invalidate the comparison. Instead, it highlights the intrinsic difficulty of interpreting facial expressions and underscores that the algorithm is more consistent and accurate than human raters on this task.

The runtime profiling on the iPhone 14 Pro showed stable resource usage across all three runs. Average overall CPU utilization ranged from 245.9% to 304.0%, while application-specific CPU usage varied between 49.6% and 99.7%. Total system memory usage remained consistent at approximately 2.0 GB, whereas the application itself required between 237 MiB and 257 MiB of memory. GPU compute durations were low, with average per-frame execution times between 525 µs and 611 µs. These results indicate that the proposed on-device pipeline operates within moderate computational and memory constraints and is feasible for real-time deployment on contemporary mobile hardware.

For comparison, processing a single 5 s video (same dataset) with OpenFace required approximately 2.5 min using CPU-only inference, which was reduced to 1.5 min with GPU acceleration. During GPU-accelerated execution, OpenFace consumed approximately 850 MiB of GPU memory, while average GPU utilization remained at around 10%. This highlights the substantially higher computational and memory demands of the deep learning baseline compared to the proposed on-device approach. Detailed data can be found in Table 4.

**Figure 4:**
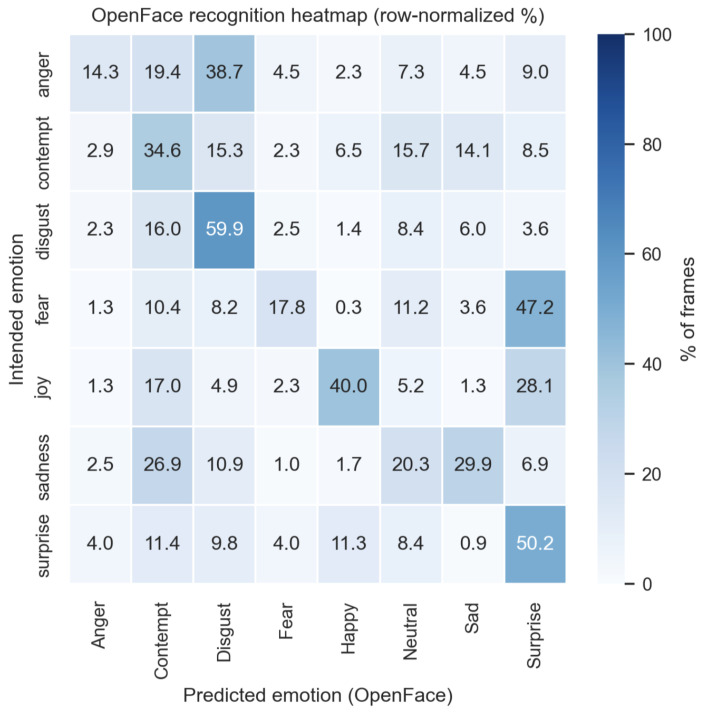
OpenFace recognition heatmap.

Figure 4 shows the row-normalized confusion matrix comparing intended emotions (y-axis) with the predictions produced by OpenFace (x-axis). Disgust was the most reliably recognized emotion, with 59.9% of frames correctly classified. Contempt achieved a moderate recognition rate of 34.6%. In contrast, anger was most frequently misclassified as disgust (38.7%).

Fear and surprise were predominantly predicted as surprise (47.2% and 50.2%, respectively), indicating substantial confusion between these categories. Sadness was correctly identified in 29.9% of frames, while a considerable proportion was misclassified as contempt (26.9%).

Notably, OpenFace frequently assigned the neutral class across multiple intended emotions, with the highest proportion observed for sadness (20.3%), suggesting a tendency of the model to favor neutral predictions in ambiguous or low-intensity expressions.

### 3.4. Per-Emotion Accuracy

Per-emotion results are listed in Table 5. Relative to baseline, cosine similarity improved markedly for disgust (+59.37 pp), fear (+71.88 pp), and joy (+59.40 pp), matched sadness, and was lower on anger and surprise. Compared to the average human, cosine similarity was higher on five of seven emotions (anger, disgust, fear, joy, and surprise), comparable on sadness (−0.61 pp), and lower on contempt (−6.15 pp).

The results in Table 5 indicate that emotions characterized by strong, stereotypical facial patterns (e.g., joy, surprise) are recognized comparatively well by both human raters and the cosine-similarity classifier. In contrast, emotions with subtle or overlapping action-unit configurations, such as contempt or the distinction between fear and surprise, show reduced accuracy for both. The classifier performs particularly well when emotions are defined by consistent multi-AU activation patterns, whereas human raters appear more variable, likely reflecting reliance on subjective interpretation and contextual expectations. These findings underscore that both human and algorithmic approaches share similar confusion patterns, while the algorithm offers higher consistency across samples.

**Figure 5:**
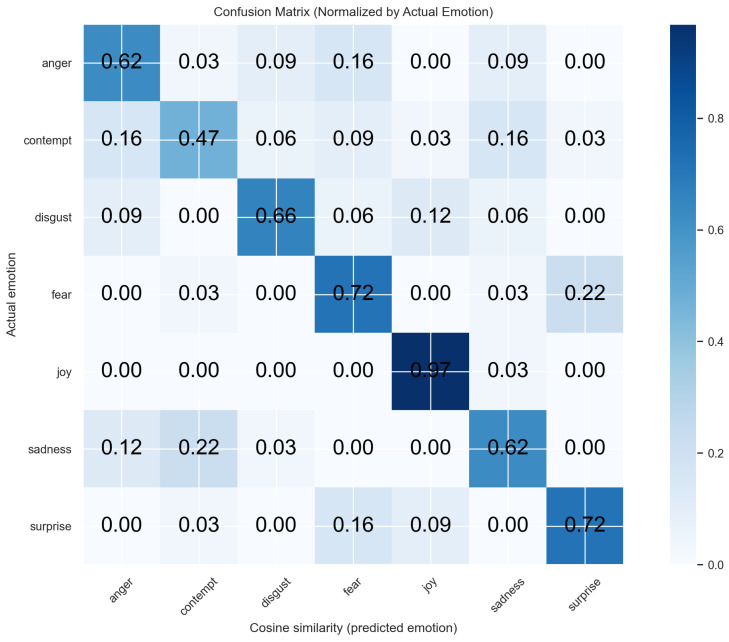
Confusion matrix for cosine similarity predicted emotions.

**Figure 6:**
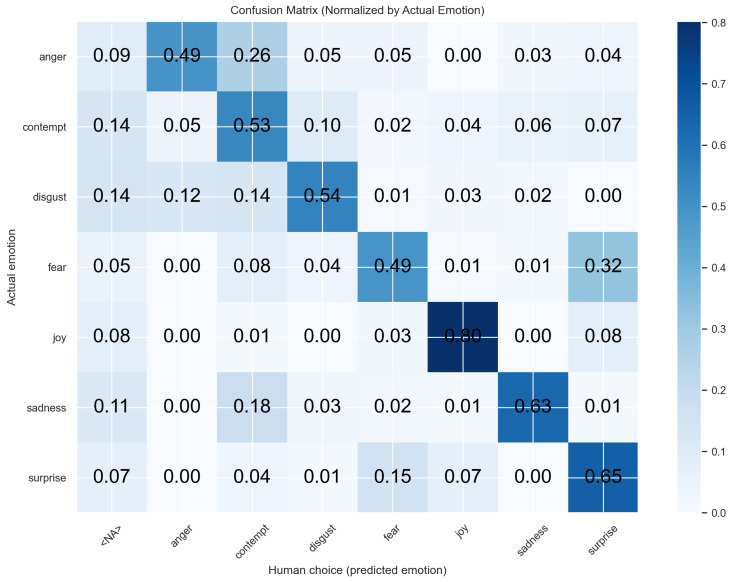
Confusion matrix for human rating predicted emotions.

In addition, we examined the confusion matrices to identify which emotions were most frequently misclassified by the human raters and by the proposed algorithm (Figure 5 and Figure 6). Overall, both humans and the algorithm exhibited similar patterns of confusion, particularly in distinguishing between surprise and fear. Notably, human raters showed a substantially higher tendency to misclassify contempt as anger than the algorithm did. This suggests that certain emotions are inherently more difficult to distinguish, for both human raters and the algorithm, likely due to overlapping blend-shape representations and the resulting similarities in their facial expressions.

### 3.5. ROC Analysis

**Figure 7:**
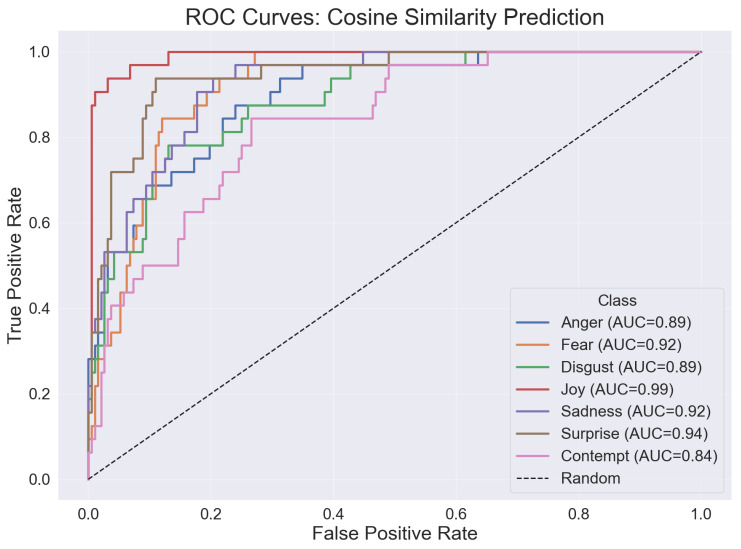
Receiver operating curve (ROC) with area under the curve (AUC) values of the cosine similarity classifier by emotion.

Receiver Operating Curve (ROC) analysis of the proposed cosine classifier demonstrated high separability across all emotions (Figure 7). AUC values were ≥0.84 for every class, with particularly strong discrimination for joy (AUC = 0.99), surprise (0.94), fear and sadness (both 0.92), and anger/disgust (0.89 each). Contempt achieved an AUC of 0.84.

### 3.6. Summary of Findings

Overall, the cosine similarity method, which is simple and scale-free, gave better accuracy than human raters on these data and did much better than a basic baseline, while the baseline performed better on anger and surprise; the new method showed large gains across other emotions and remained consistently accurate, demonstrating its effectiveness for real-time, on-device emotion detection.

## 4. Discussion

### 4.1. Key Findings

This work demonstrates that a lightweight cosine-similarity classifier operating directly on ARKit blend shape vectors can deliver good real-time emotion recognition performance. Without any model training, feature learning, or hyperparameter tuning, the proposed approach achieved an overall accuracy of 68.31% and high per-class separability (AUCs ≥ 0.84, peaking at 0.99 for joy). Notably, it surpassed the average human rater (58.86%) and the best individual rater (64.75%). These findings support the central premise that a scale-invariant, computationally inexpensive metric is well-suited for real-time, on-device inference, where memory, energy, and latency budgets are constrained.

Cosine similarity exploits the directional information of standardized blend shape vectors, emphasizing relative activation patterns rather than absolute magnitudes. After subject-wise z-scoring, this yields invariance to individual baselines and expression intensity, which likely contributed to robust discrimination for expressions defined by characteristic co-activations (e.g., joy: smiling; fear/surprise: eye widening and jaw opening). The consistently high AUCs indicate that decision thresholds can be tuned to prioritize sensitivity or specificity across different deployment contexts without redesigning the algorithm.

While the proposed method improved markedly over the baseline (mean-of-sums) approach for most categories, two classes (anger, surprise) were stronger under the baseline. This pattern suggests that for some expressions, a small subset of highly discriminative action units may dominate (e.g., AU4 for brow lowering in anger), making aggregate intensity a useful proxy. Conversely, emotions such as fear, disgust, and joy benefitted from pattern-level matching across multiple action units, where cosine similarity excels. The lower performance for contempt is consistent with known difficulties: contempt is often expressed unilaterally, can be subtle, and is easily confused with neighboring affective states. In practice, a hybrid strategy that combines pattern similarity with targeted single-AU checks may close the residual gap for these edge cases.

Human raters provide an informative baseline but are themselves variable. In our data, the psychologists did not agree perfectly with each other nor with the third rater, underscoring that labeling facial expressions—even when posed—remains challenging. Inter-rater reliability (e.g., Kirpendorff’s α) offers a principled way to contextualize machine performance relative to the variability of human annotations. Taken together with accuracy, such agreement metrics help determine whether a system is human-like in its decisions or consistently better aligned with a specific rater consensus. The low inter-rater agreement among human raters in our study underscores that this task is inherently difficult, even for trained observers. As a consequence, we do not interpret average human accuracy as a strict performance baseline. Rather, human ratings are included as a side-by-side comparison to contextualize algorithmic performance and to demonstrate that consistent interpretation of facial expressions cannot be assumed. From this perspective, the proposed method’s consistency and reproducibility highlight the potential value of computational approaches as decision-support tools rather than replacements for human judgment.

The method’s simplicity has concrete sensor-level advantages. First, inference is O(d) in the number of blend shapes, with no model parameters to store and a negligible memory footprint. Second, privacy is improved when all computation occurs locally, and only anonymized summaries or decisions need to be persisted. Third, deployment is straightforward across device generations because cosine similarity relies on the relative geometry of features rather than exact numeric ranges. Finally, interpretability is preserved as predicted labels can be traced to the contributing blend shapes, which aids debugging and human factors evaluation.

A qualitative inspection of the confusion matrices reveals systematic error patterns that can be explained by overlapping facial action-unit configurations. In particular, fear and surprise are frequently confused by both the algorithm and human raters, which is consistent with their shared reliance on eye widening and jaw opening. These overlaps are visible in the blend shape activation patterns shown in Figure A1 and in the co-activation of eye and jaw regions, limiting separability based on facial geometry alone. Contempt presents a distinct challenge as it is often expressed subtly and asymmetrically, commonly involving unilateral AU14 activation. In our implementation, symmetric aggregation of left and right blend shapes likely attenuates such asymmetric cues, contributing to reduced recognition performance. Preserving asymmetry for selected blend shapes therefore represents a promising avenue for future improvement. Notably, emotions associated with higher algorithmic confusion also show low inter-rater agreement among human observers. This correspondence indicates that misclassifications largely reflect intrinsic ambiguity in the expressions rather than purely methodological limitations.

Unlike learned emotion classifiers, our approach requires no training and avoids bias from training data, class imbalance, or overfitting to specific faces or demographics. Any regular error can only come from ARKit’s facial shape system, which we do not control. This separation is useful, as errors can be traced to the sensor itself rather than the method, allowing us to judge directly how useful the facial shape data alone is.

### 4.2. Comparison with Prior Work

Our approach sits at the intersection of classical FACS/EMFACS-driven analysis and modern, on-device sensing. FACS taxonomy and its EMFACS mapping have long underpinned algorithmic facial emotion recognition by linking AUs to discrete emotions and are used widely for facial behavior analysis. In contrast, some studies have focused on extracting expressions from depth information, whereas our study relies on off-the-shelf sensors and existing frameworks (ARKit), paralleling the research by Johanssen et al. but differing by including a comprehensive, in-depth performance analysis. This contrast emphasizes our focus on standard device compatibility and more complete benchmarking, distinguishing the scope of our study from prior depth-based efforts.

While much recent work emphasizes emotion recognition in naturalistic or “in-the-wild” settings, this study adopts a controlled elicitation paradigm motivated by medical and methodological considerations. In clinical contexts, reproducibility, interpretability, and well-defined ground truth are often prerequisites for validation before deployment in less constrained environments. By first establishing performance under controlled conditions, we aim to provide a reference point for subsequent studies that extend on-device emotion recognition to naturalistic scenarios.

From a sensing standpoint, our work exploits blend shape coefficients that ARKit streams in real-time from the TrueDepth front camera. Similar coefficient streams are also exposed by alternative stacks (e.g., MediaPipe Face Landmarker), suggesting cross-framework portability of our cosine-similarity recipe and making it attractive for embedded applications where latency and energy are first-order constraints. Our results indicate that these same coefficients, combined with EMFACS-informed prototypes, are sufficient for accurate emotion detection without the need for deep models.

A direct comparison with our findings reveals both overlaps and divergences. Similar to Geraets et al. [20], we observed high recognizability for surprise and moderately strong performance for anger/“rage,” although in our case the cosine-similarity method did not surpass human performance for surprise. Conversely, our classifiers—and to a lesser extent the human raters—showed comparatively higher accuracy for joy and disgust than reported in Geraets et al.’s virtual-reality setting [20], while fear and sadness followed a more similar pattern across both studies. These differences likely reflect distinct elicitation protocols (posed vs. task-evoked expressions), sensor modalities, and the mapping between AU patterns and discrete labels. Overall, while both studies highlight the variability in human emotion recognition, our results demonstrate that a simple cosine-similarity approach can achieve competitive or superior accuracy across several emotion categories using ARKit’s blend-shape representation. In this context, our cosine approach (overall accuracy 68.3%) exceeds the mean human accuracy (58.9%) and two individual raters and approaches the best rater, exemplifying the value of simple, geometry-based similarity measures for real-time emotion recognition.

### 4.3. Practical Considerations

For reliable real-time emotion recognition using blend shape-based approaches, several practical factors should be considered. First, the face should be clearly visible to the camera, with a moderate and stable distance. In our setup, a distance of approximately 30–40 cm yielded robust tracking. Adequate and uniform lighting, as well as stable handling or mounting of the device, further improve tracking quality. Second, device capabilities matter, as newer devices with higher processing power and more stable sampling rates are likely to provide more consistent performance, although this was not systematically evaluated in the present study. Third, while our implementation uses Apple’s ARKit, similar frameworks that provide blend shape representations (e.g., MediaPipe) could serve as alternatives, and the cosine-similarity-based classification approach should transfer naturally to such representations. Together, these considerations provide practical guidance for applying and extending the proposed method.

### 4.4. Limitations

Despite these contributions, this study has several limitations that constrain the generalizability of its findings. First, the dataset was obtained via convenience sampling (*N* = 31), predominantly from staff and students, and expressions were posed rather than spontaneous. These design choices were made to establish a reproducible and interpretable benchmark for on-device emotion recognition, but they do not capture the full variability of spontaneous facial behavior encountered in real-world or clinical settings.

Second, all data were captured on a single device (iPhone 14 Pro) under good lighting with mostly frontal poses. As a result, robustness to different cameras and firmware, head poses, occlusions (e.g., masks, hands), and illumination changes remains untested. Class characteristics also matter: some emotions, notably contempt, are subtle and often unilateral (e.g., AU14). Our aggregation of left/right movements may blur diagnostic asymmetry cues and depress performance in such cases.

A further limitation concerns the validity of ARKit’s AU-inspired blend shapes. These coefficients do not always map cleanly onto their intended FACS units, and some exhibit unexpected co-activations (e.g., noseSneer with cheekSquint). This implies that emotion prototypes derived from EMFACS inherit any inaccuracies in ARKit’s underlying AU representation, motivating future work to validate and, if necessary, refine these mappings before addressing higher-level affect classification.

Third, frame-rate variability was present: recordings occurred at both 60 and 30 FPS. We standardized by downsampling 60 FPS sequences to 30 FPS to avoid upsampling artifacts, but this may discard micro-dynamics that are informative for recognition. The cause of the FPS inconsistency is unclear; thermal throttling is a plausible explanation (https://stackoverflow.com/questions/53569220/arkit-randomlylimits-frame-rate-to-30fps (accessed on 16 June 2025)) although we could not verify this.

Fourth, timing precision was imperfect for the first 12 subjects because app-generated timestamps were only accurate to the nearest second. We reconstructed finer timing by combining the initial timestamp with frame counts to approximate per-frame times. This procedure reduces, but does not eliminate, small temporal errors.

Fifth, human labels contain substantial noise. Inter-rater agreement in our dataset was extremely low (Krippendorff’s α=0.01), indicating that raters often diverged in how they interpreted the same expression, even when the underlying ground truth was objectively defined by the instructed emotion. Consequently, accuracy against these human labels is an imperfect proxy for true affect and should be interpreted in the context of this annotation variability. Reporting model performance alongside inter-rater reliability metrics clarifies that part of the apparent performance gap reflects inconsistency among raters rather than deficiencies in the classifier itself.

Our evaluation focused on seven discrete basic emotions commonly operationalized via EMFACS/Ekman-style mappings, and while this choice enables clear, reproducible action-unit (AU) definitions and a controlled benchmark, clinical facial behavior is not limited to these categories and often includes mixed, subtle, context-dependent, or symptom-relevant affective states. Extending on-device recognition beyond the basic set requires additional methodological work, in particular, establishing clinically meaningful target states and their corresponding AU/blend-shape definitions (including intensity, laterality, and co-activation patterns). Therefore, our results should be interpreted as a first step demonstrating feasibility on commodity hardware rather than a complete coverage of clinically relevant facial expressions.

Finally, subject selection limits demographic and clinical coverage. The sample was predominantly Caucasian, with only a few participants of Arabic and Asian backgrounds. All participants were healthy; however, not all expressed emotions within a normative range, and one subject may have produced atypical expressions, as judged by a trained psychologist during video review. Populations with neurological conditions (e.g., Parkinson’s disease, frontotemporal dementia) may display altered facial expressivity and timing, and performance of ARKit+FACS in those groups may differ from the results reported here.

### 4.5. Future Work

Building on these findings, several steps can strengthen external validity and practical deployment. The dataset should be scaled and diversified by increasing sample size, expanding demographic coverage, and including clinical cohorts (e.g., Parkinson’s disease, frontotemporal dementia). A balanced design across emotions, head poses, lighting, and occlusions would reduce confounding and enable stratified analyses with confidence intervals.

Methodologically, lightweight personalization is a promising next step. Updating emotion centroids with a few subject-specific examples and using longer neutral segments as baselines may reduce inter-individual differences. Temporal cues can be added with minimal complexity—for example, short-window cosine similarity, exponential smoothing, or simple frame voting. For emotions tied to distinctive action units (e.g., AU4 for anger, AU14 for contempt), combining cosine similarity with targeted AU rules could improve recognition. Given the consistently high AUCs, decision thresholds can be tuned to application needs (e.g., sensitivity for assistive use or specificity for clinical settings). Future work should include a more systematic error analysis linking misclassification patterns to overlapping and asymmetric blend shape activations.

Data acquisition and instrumentation can also be improved. More precise timestamps, stable frame rates (ideally 60 FPS), and logging device temperature or system load would help separate temporal effects from hardware throttling. Retaining left–right asymmetry for selected blend shapes may aid recognition of unilateral expressions such as contempt. Evaluation should also report inter-rater reliability, human–machine agreement, and uncertainty (e.g., bootstrap intervals) to contextualize accuracy.

Future work should extend the framework to clinically relevant facial expressions beyond the basic emotions, including mixed affect and condition-specific facial behaviors. This will require the following: (i) defining target constructs and ground truth in a clinically valid way (e.g., AU-based definitions, intensity scales, and temporal profiles), (ii) validating or adapting AU/blend-shape mappings for these constructs, and (iii) collecting appropriately annotated datasets in the intended populations. Such steps are necessary before broader clinical claims can be made for non-basic emotion categories.

Additionally, future work should evaluate the approach on established facial expression datasets by re-tracking existing videos with compatible face-tracking frameworks or by collecting benchmark datasets that provide standardized blend shape representations, as most current datasets do not directly support ARKit-style blend shapes. Another direction for future work is to apply the cosine-similarity approach to other facial analysis frameworks and datasets, such as MediaPipe or AffectNet, to assess cross-framework generalizability.

Finally, cross-framework and in-the-wild validation are essential. Replicating the protocol with alternative trackers (e.g., MediaPipe) and across device generations will clarify whether gains stem from expression geometry rather than platform-specific features. Moving beyond posed expressions to conversational or naturalistic scenarios will test the approach under real conditions. These steps aim to preserve the simplicity of the real-time cosine classifier while improving its reliability, equity, and portability across users, sensors, and use cases.

## 5. Conclusions

A cosine-similarity classifier on standardized ARKit blend shapes offers an attractive accuracy–complexity trade-off for embedded real-time emotion sensing. In line with the research goals stated in the Introduction, this study (i) described and evaluated a fully on-device, interpretable emotion-recognition pipeline based on FACS-guided prototypes and cosine similarity that requires only per-frame blend shape vectors and no server-side processing, (ii) established and reported a native iOS/TrueDepth measurement protocol to elicit 36 blend-shape-targeting movements and seven discrete emotions in N=31 participants, and (iii) to contextualize the proposed method’s performance with three independent human raters using the same video material. Across all emotions, the cosine-similarity approach achieved an overall accuracy of 68.3% and AUCs ≥ 0.84 for all classes, outperforming the mean human accuracy (58.9%) and substantially exceeding a simple mean-based baseline (40.2%). The proposed method achieves performance comparable to, and in some cases exceeding, individual human raters on this task, while providing substantially higher consistency. These findings suggest that algorithmic support may be particularly valuable in settings where human interpretation of facial expressions is variable or unreliable. Together, these results confirm that our goals were realized: a simple, interpretable, real-time method operating entirely on-device can perform better than humans while using commodity hardware, supporting privacy-preserving mobile applications. With targeted improvements (personalization, temporal context, and hybrid AU checks) and broader validation across devices and naturalistic settings, cosine-based pattern matching provides a practical foundation for real-time, on-device emotion detection. While these results demonstrate the feasibility of real-time, on-device emotion recognition under controlled conditions, their generalizability to diverse populations and spontaneous, in-the-wild expressions remains to be established.

## Figures and Tables

**Table 1:** Analyzed blend shapes for each emotion.

Blend Shape	Anger	Contempt	Disgust	Fear	Joy	Sadness	Surprise
browDownLeft	4		4	4		4	
browDownRight	4		4	4		4	
browInnerUp				1		1	1
browOuterUpLeft				2			2
browOuterUpRight				2			2
cheekSquintLeft					6		
cheekSquintRight					6		
eyeSquintLeft	7			7			
eyeSquintRight	7			7			
eyeWideLeft	5			5			5
eyeWideRight	5			5			5
jawOpen				26			26
mouthDimpleLeft		(*) 14					
mouthDimpleRight		(+) 14					
mouthFrownLeft			15			15	
mouthFrownRight			15			15	
mouthLowerDownLeft			16				
mouthLowerDownRight			16				
mouthPucker	23						
mouthSmileLeft		(*) 12			12		
mouthSmileRight		(+) 12			12		
mouthStretchLeft				20			
mouthStretchRight				20			
noseSneerRight			9				
noseSneerLeft			9				

For contempt, only one side of the face is active: either both (*) and not (+) or both (+) and not (*). The blend shapes that are not used in any emotion recognition are as follows: eyeBlinkLeft, eyeBlinkRight, eyeLookDownLeft, eyeLookDownRight, eyeLookInLeft, eyeLookInRight, eyeLookOutLeft, eyeLookOutRight, eyeLookUpLeft, eyeLookUpRight, tongueOut, jawLeft, jawRight, cheekPuff, mouthUpperUpRight, mouthUpperUpLeft, mouthShrugUpper, mouthShrugLower, mouthRollUpper, mouthRollLower, mouthFunnel, mouthPressRight, mouthPressLeft, mouthRight, mouthLeft, mouthClose, jawForward.

**Table 2:** Overlap of opposite blend shapes, indicating the amount of data points where both blend shapes are activated at the same time.

blendshape_1_	blendshape_2_	Overlap
browDownLeft	browOuterUpLeft	0.00
browDownRight	browOuterUpRight	0.00
eyeLookDownLeft	eyeLookUpLeft	0.00
eyeLookDownRight	eyeLookUpRight	0.00
eyeLookInLeft	eyeLookOutLeft	0.00
eyeLookInRight	eyeLookOutRight	0.00
eyeWideLeft	eyeSquintLeft	0.25
eyeWideRight	eyeSquintRight	0.25
mouthFrownLeft	mouthSmileLeft	0.00
mouthFrownRight	mouthSmileRight	0.00
mouthLeft	mouthRight	0.00
jawLeft	jawRight	0.00

**Table 3:** Overall accuracy across methods and human raters.

Method/Rater	Accuracy
Baseline (Sum of Relevant Blend Shapes)	40.18%
Cosine Similarity (Proposed)	68.30%
Human–R1	59.66%
Human–R2	64.75%
Human–R3	52.17%
Human–Average	58.86%

**Table 4:** CPU, memory, and GPU performance metrics across three runs.

Metric	Run 1	Run 2	Run 3
Overall System CPU Usage (%)			
Average	250.8	304.0	245.9
Min–Max	220.9–296.1	236.2–358.9	191.9–310.9
Overall System Memory Usage (GB)			
Range	1.98–2.02	1.97–2.01	1.97–2.03
Application CPU Usage (%)			
Average	52.9	99.7	49.6
Min–Max	41–67	87–109	40–64
Application Memory Usage (MiB)			
Average	237.11	256.95	237.08
GPU Compute Duration (μs)			
Average	610.63	524.52	593.35

**Table 5:** Per-emotion accuracy: baseline vs. cosine similarity (proposed) vs. average human.

Emotion	Baseline	Proposed	Human Avg	Δ (Prop−Human)
Anger	75.00%	62.50%	49.02%	+13.48%
Contempt	18.75%	46.88%	53.03%	−6.15%
Disgust	6.25%	65.62%	54.22%	+11.4%
Fear	0.00%	71.88%	48.59%	+23.29%
Joy	37.47%	96.87%	80.20%	+16.67%
Sadness	62.51%	62.51%	63.12%	−0.61%
Surprise	81.25%	71.88%	65.22%	+6.66%

## Data Availability

The data from this study can be provided anonymously upon reasonable request from the corresponding author.

## Abbreviations

The following abbreviations are used in this manuscript:

FACS	Facial Action Coding Systems
AU	Action Units
BMI	Body mass index
EMFACS	Emotional Facial Action Coding System
ROC	Receiver Operating Curves
AUC	Area Under the Curve
